# Comparing Sexual and Gender Minority and Cisgender Heterosexual Missourians’ Breast and Colorectal Cancer Screening Prevalence: The 2022 Missouri County-Level Study

**DOI:** 10.3390/cancers18050729

**Published:** 2026-02-24

**Authors:** Jane A. McElroy, Kevin D. Everett

**Affiliations:** Family and Community Medicine Department, University of Missouri, Columbia, MO 65211, USA; everettk@umsystem.edu

**Keywords:** breast cancer screening, colorectal cancer screening, prevalence, sexual and gender minority (SGM) populations

## Abstract

Background: Cancer screening disparities remain understudied, particularly among underrepresented groups at the county level. This study compared the use of preventive breast and colorectal cancer screening services between sexual and gender minority (SGM) adults and cisgender heterosexual adults in Missouri. Methods: The 2022 Missouri County-Level Study, a probabilistic survey of health-related behaviors in each county, was used to estimate breast and colorectal cancer (BC and CRC) screening prevalence. Screening prevalence was calculated using weighted samples, and regression models were used to adjust for demographic composition and age eligibility for both cancer sites. Results: Compared to cisgender heterosexual adults (n = 48,257), SGM adults (n = 2801) were significantly younger and more likely to reside in urban areas and be employed. Statewide, county-representative prevalence of breast cancer screening in the last 2 years was 75.6%, and colorectal cancer screening (i.e., colonoscopy in the last 10 years or sigmoidoscopy in the last 5 years) was 63.1%. In age-adjusted models for BC screening for participants (ages 40–74), age had a curvilinear association, increasing at younger ages but declining in later years. For CR screening (ages 45–75), age showed a strong, stable, positive effect. SGM adults had similar odds of breast cancer screening; however, for CRC, SGM adults had higher odds of ever being screened but similar odds to cisgender heterosexual adults of up-to-date screening. Differences largely reflect eligibility windows and initiation versus maintenance dynamics. Conclusions: In this large sample of Missouri county residents, breast cancer and colorectal cancer screening rates were comparable between SGM adults and cisgender heterosexual adults for up-to-date screening probability. Improving statewide cancer prevention will require addressing the broader structural and regional barriers that suppress screening uptake across Missouri communities. Impact: These findings demonstrate the importance of using age-appropriate, guideline-aligned analyses to accurately assess cancer screening equity and avoid overstating disparities among SGM populations. By identifying where differences do not exist, this work helps focus resources on the structural and regional barriers that continue to limit cancer prevention for all Missourians.

## Simple Summary

This study explores whether adults who identify as sexual and gender minorities (SGM) in Missouri are receiving recommended breast and colorectal cancer screening at similar rates as cisgender heterosexual adults. Regular breast and colorectal cancer screenings can help find cancer early, yet little research has examined how screening rates differ for underrepresented groups at the local level. Using a statewide, county-representative survey, the researchers compared how often SGM adults and cisgender heterosexual adults completed mammograms and colonoscopies. The results showed no significant differences in breast or colorectal cancer screening between SGM adults and cisgender heterosexual adults once analyses were limited to people who were eligible for screening based on age. Although models including all adults suggested lower mammography use among SGM women, this pattern disappeared after adjusting for screening-eligible ages. These findings show that age composition, not reduced access, explains the observed differences and highlight the importance of using guideline-based age groups when assessing cancer screening equity. Understanding true screening patterns can help ensure that prevention efforts remain inclusive and appropriately targeted across Missouri’s diverse communities.

## 1. Introduction

According to the National Center for Health Statistics, cancer is the second leading cause of death in the United States [1]. Reducing cancer morbidity and mortality requires addressing modifiable behavioral risks (e.g., tobacco and alcohol use) and improving uptake of preventive screening services, including breast cancer screening via mammography and colorectal cancer screening via colonoscopy. Removal of precancerous polyps during a colonoscopy can prevent colorectal cancer [2]. The value of routine breast and colorectal cancer screenings lies in the reduction in cancer mortality from 14 to 65%, thereby saving lives [2,3,4,5,6]. The Healthy People 2030 goal for breast cancer (BC) screening is 80.3%, and in 2021, 75.6% of women aged 50–74 years had been screened [7]. For CRC screening among people aged 45–75 years, the Healthy People 2030 goal is 68.3%, and in 2021, 58.7% had been screened [8]. In Missouri, the estimated breast and colorectal cancer screening prevalences in 2020, the most recent year of data, were 70% and 73%, respectively [9].

Patient- and clinician-level barriers strongly influence screening behaviors [10,11,12,13]. Studies indicate that patients who face socioeconomic challenges, such as those who are underinsured, with no insurance, or low income, have lower BC and CRC guideline-concordant cancer screening rates [10,11,12,13]. Scheduling difficulties, fear, embarrassment, language barriers, and lack of knowledge of the importance of screening are other commonly reported barriers to screening [14,15]. Clinic- and clinician-level barriers to screening include ineffective communication with patients, competing demands during a clinic visit, inadequate prioritization and resources dedicated to screening, and poor electronic health record integration of evidence-based interventions to support screening [12,16].

Limited information is available on the SGM population’s BC and CRC guideline-concordant cancer screening prevalence since sexual orientation and gender identity are not routinely collected at procedural sites for BC or CRC screening. Results from a few surveillance studies find cancer screening rates to be lower for SGM adults compared to cisgender heterosexual adults [17,18]. SGM populations, in addition to the described barriers faced by all patients, likely face additional barriers. A significant portion of the SGM community reports avoiding medical care due to perceived discrimination by medical clinicians [19]. Further, poor communication by clinicians who lack cultural competence is cited as a barrier to cancer screening for SGM patients [20]. The minority stress model posits that SGM groups can experience internal and external stressors leading to higher rates of mental distress and maladaptive coping [21,22]. This is borne out in higher cancer risk behaviors in SGM (e.g., smoking and alcohol use) and lower utilization of healthcare [23,24,25,26,27,28]. The purpose of this study is to estimate the prevalence of BC and CRC screening between SGM adults and cisgender heterosexual adults and determine how age eligibility and demographic factors shape screening patterns using data from the Missouri County-Level Study (MO-CLS).

## 2. Materials and Methods

We conducted a descriptive cross-sectional study using county-level data from the 2022 MO-CLS. This dataset provides comprehensive information on health behaviors and outcomes across Missouri counties. Our analysis focused on estimating the prevalence and identifying predictors of BC and CRC screening among Missouri residents.

Missouri comprises 114 counties and the City of St. Louis. The MO-CLS collects data about Missouri residents’ health-related risk behaviors, chronic diseases and conditions, health care access, and use of preventive services from each county every 5 years. This descriptive cross-sectional study provides data to determine the county-level prevalence. MO-CLS follows the established study design used by the US Centers for Disease Control and Prevention (CDC) annual Behavioral Risk Factor Surveillance System (BRFSS) study [29,30]; however, the sampling frame is different. The 2022 MO-CLS sampled residents from every Missouri county and the City of St. Louis, providing county-level representation. Unlike the BRFSS, which uses a statewide random-digit-dial (RDD) sample designed primarily to produce state-level estimates and regional pooled estimates, MO-CLS employs a county-stratified sampling frame with predetermined interview targets for each county. A minimum of 300 interviews were conducted in most counties, substantially oversampling rural and less populated areas compared with BRFSS, while more populous counties (e.g., Boone, Jackson, St. Louis County, and the City of St. Louis) received larger samples to support subgroup analyses by race/ethnicity and rural/urban residence. In practical terms, the BRFSS sampling probability is proportional to the statewide distribution of landline and cell numbers, whereas MO-CLS assigns sampling quotas to each county regardless of its population size, ensuring adequate representation of smaller rural counties that would otherwise have insufficient sample sizes under a standard BRFSS design [30,31,32]. Data were weighted to reflect the noninstitutionalized adult population of each county using iterative proportional fitting (ranking). The Missouri Department of Health and Senior Services (DHSS) follows the Common Rule (45 CFR 46) when conducting human subjects research as part of routine public health surveillance. Under 45 CFR 46.102(I)(2), public health surveillance activities conducted by a public health authority are not considered human subject research. Therefore, IRB review and approval were not required.

### 2.1. Measures

Items in the MO-CLS consist of BRFSS and CDC Adult Tobacco Survey items. For SGM status, gender identity (GI) was defined as a response to the question: Do you consider yourself to be transgender? The answer options were as follows: (1) yes, transgender, male-to-female; (2) yes, transgender, female-to-male; (3) yes, transgender, gender nonconforming; and (4) no. An SGM status of “yes” included answer options 1–3. Sexual orientation (SO) was defined as a response to the question: Which of the following best represents how you think of yourself? The answer options were as follows: (1) lesbian or gay; (2) straight, that is, not gay; (3) bisexual; and (4) something else. No additional information was gathered about those who were marked as “something else” by the interviewer. A response of “yes” to answer options 1, 3, and 4 was considered to identify SGM participants. To be defined as a cisgender heterosexual adult, the participant marked “no” for the GI question and marked “straight, that is, not gay” for the SO question. Male or female was defined as “what was your sex at birth; was it male or female?” Participants who did not know or refused to answer were not included.

Participant characteristics included age, urban–rural status (4 rural–urban commuting area (RUCA) categories: urban, large rural, small rural, and isolated) [33], race/ethnicity (5 categories: non-Hispanic (NH) White, NH African American, NH multiracial, NH another race, Hispanic), education (2 categories: <bachelor’s degree, bachelor’s degree or other advanced degree), income (5 categories: <$25K, $25K–49,999, $50K–74,999, $75K–99,999, $100K+), employment (4 categories: employed, retired, unemployed/homemaker/student, unable to work), insurance status (3 categories: employer (private), Medicare/Medigap, Medicaid or other assistance), home ownership (y/n), exercise in last 30 days (y/n), smoking status (3 categories: never, former, current), and marital status (3 categories: single/never married, married, divorced/widowed/separated).

Colorectal cancer (CRC) screening used two sets of yes/no questions: (1) if the individual has ever had a colonoscopy or sigmoidoscopy, and (2) if the individual has received a colonoscopy within the past 10 years or received a sigmoidoscopy within the past 5 years. Breast cancer (BC) screening also used two questions: (1) if the individual has ever had a mammogram, and (2) if the individual has had a mammogram within the past 2 years. To be considered “timely screening,” both questions for each cancer had to be marked in the affirmative. BC screening questions were asked of all female respondents aged 18 to 99 years; all respondents aged 45 to 99 years were asked CRC screening questions.

### 2.2. Analytic Plan

Initial descriptive statistics were used to characterize the sample, and chi-square tests assessed differences between SGM adults and cisgender heterosexual adults. Cramér’s V was calculated to assess the strength and practical significance of associations. Effect size interpretation followed conventional thresholds, where values below 0.06 were considered weak, values of 0.06–0.17 were considered small-to-moderate, and values above 0.17 were considered moderate [34]. BC and CRC screening rates were mapped at the county level using three categories: below, at, and above the average state screening rates.

Using SAS 9.4 software (Cary, NC, USA), a series of regression models was developed to examine predictors of screening completion for BC and CRC. Age was treated as a continuous variable. For both BC and CRC screening, prevalence by age was estimated separately for the following: (1) having ever completed a screening and (2) having completed the most recent screening within the guideline-recommended [35,36,37,38] interval, thereby remaining compliant. These two prevalence estimates were then multiplied to calculate a final joint probability of completing a “timely screening” at each age. Two screening outcomes were evaluated. “Ever screened” captured lifetime mammography or colonoscopy/sigmoidoscopy access and cumulative screening opportunities. “Screened within the past two years” for breast cancer and “screened within the past 10 years for colonoscopy and 5 years for sigmoidoscopy” reflected adherence to current USPSTF guidelines and recent preventive care engagement. Including both outcomes served as a sensitivity analysis to determine whether associations with SGM status were robust to different operational definitions of screening. Covariance matrices were used to evaluate all regressors for variability and collinearity. Primary analyses were restricted to screening-eligible age ranges (breast: 40–74; colorectal: 45–75). As a sensitivity/descriptive check, we repeated models in the full adult sample (breast: 18–99; colorectal: 45–99). These results are reported in Supplementary Tables S1 and S2.

Because the Missouri Cancer and Lifestyle Survey (MO-CLS) uses a BRFSS-like sampling approach, sampling weights (_llcpwt) were incorporated into all regression models and probability estimates to ensure population-representative results. Complete and consistently available stratum and cluster identifiers were not provided for all analytic subsets; therefore, full Taylor-series or replicate-weight variance estimation could not be applied uniformly across models [39]. Weighted regression models were used to generate individual model-estimated probabilities (MEPs), and weighted means were used to summarize final BC screening and CRC screening MEP values at the population level. Sensitivity checks comparing weighted and unweighted standard errors yielded highly similar results, and all substantive conclusions from the primary age-eligible analyses remained unchanged.

Three sets of regression models were developed to comprehensively evaluate screening status by age and demographics. First, probit or generalized linear models (GLMs) were used to estimate the probability of ever having completed the screening (e.g., “Have you ever had a mammogram?” for BC or “Have you ever had a colonoscopy or sigmoidoscopy?” for CRC). Second, for individuals responding “Yes,” additional nonlinear quadratic regressions modeled the probability that the most recent screening occurred within the recommended time interval [35,36,37,38] (2 years for mammography; 10 years for colonoscopy or 5 years for sigmoidoscopy). These models incorporated age as a key continuous predictor along with linear demographic effects. In the tables, negative estimates are shown in parentheses. Finally, the joint probability of “timely screening” at any given age was calculated as the product of the probabilities obtained from the first two models. Models used data for guideline-concordant ages (BC: 40–74 years and CRC: 45–75 years) and all ages (results reported in Supplementary Tables). The distribution of point estimates for timely screening was visualized by status using histograms and whisker plots.

## 3. Results

**Figure 1:**
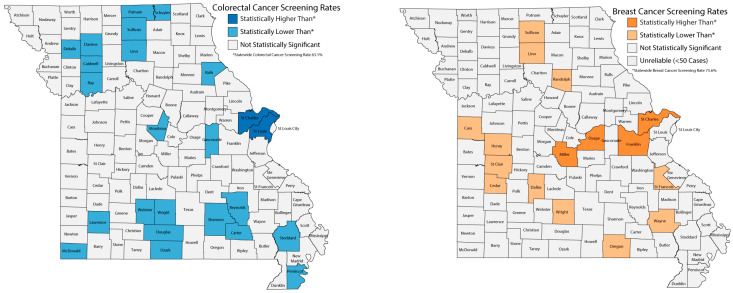
Breast and colorectal cancer screening rates by county, 2022 county-level data.

The MO-CLS garnered 50,206 completed surveys in 2022 with an overall survey response rate of 59%. The overall prevalence of BC screening was 75.6%, with 75.8% for cisgender heterosexual women and 71.5% for SGM women. Five counties had significantly higher mammography screening rates than the state average, while 12 counties had significantly lower rates. All but one of these counties were rural (Figure 1). The overall CRC screening rate across Missouri counties was 63.1%, with 63.3% for cisgender heterosexual adults and 60.3% for SGM adults. Two urban counties had significantly higher CRC screening rates than the state average, while 22 counties had significantly lower rates, all of which were rural (Figure 1).

When comparing the groups, SGM adults are significantly younger (66% under 40 years vs. 35% under 40 years), more likely to have never been married (55% vs. 25%), lived in urban areas (81% urban vs. 74%), and smoked (19% vs. 15%). They were less likely to be identified as NH White (77% vs. 80%), have earned $100,000 or more (19% vs. 30%), be retired (8% vs. 22%), utilize Medicare/Medigap (11% vs. 23%), and own their home (48% vs. 71%) (Table 1). Among SGM adults, 63% were female at the full-sample level (n = 2801); within the screening-eligible ages, 55% were female for ages 40–74 (n = 1069) and 56% for ages 45–75 (n = 905).

Chi-square tests indicated statistically significant differences between groups as noted above, primarily due to large sample sizes. Effect size estimates using Cramér’s V revealed that most associations were weak or extremely weak, suggesting minimal practical differences. Notable exceptions included age groups (V = 0.173), employment status (V = 0.105), marital status (V = 0.181), and home ownership (V = 0.131), which demonstrated small-to-moderate associations and may represent more meaningful differences (Table 1).

**Figure 2:**
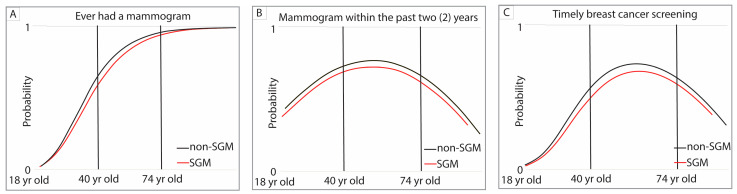
Probability of ever having a mammogram (**A**), in the past two years (**B**), and timely breast cancer screening (**C**) for SGM adults and non-SGM adults.

The question “Have you ever had a mammogram?” was asked of 27,520 Missouri women aged 18 to 99 years. Missing demographic information reduced the analytic sample to 17,815 and 10,062 women aged 40–74 years who reported receiving BC screening in the last 2 years. The age-based prevalence function for a “Yes” response followed a sigmoidal shape, with near-zero probability at age 18, rising sharply within the recommended screening age range of 40 to 74 years [35,36,37,38], and tapering toward a probability of one at the end of life for both SGM women and cisgender heterosexual women. This pattern suggested that nearly every Missouri woman would receive at least one mammogram in her lifetime (Figure 2A).

The probability of reporting a mammogram within the past two years, plotted by age, among SGM women and cisgender heterosexual women, showed a rise in screening probability up to midlife (approximately ages 50–60), then a decline as they approached age 74. SGM women exhibited consistently lower screening probabilities than their cisgender heterosexual counterparts across the guideline-recommended age range [35,36,37,38] (Figure 2B).

The probability of timely breast cancer screening, shown by age, followed a similar pattern to recent mammography: both SGM and cisgender heterosexual women showed rising screening probability into midlife (around ages 50–60) and a gradual decline thereafter. Across the guideline-recommended screening ages of 40–74, SGM women consistently demonstrated lower probabilities of receiving timely breast cancer screening compared with cisgender heterosexual women (Figure 2C).

**Figure 3:**
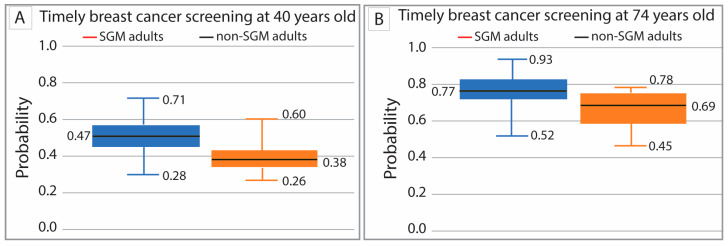
Whisker plot of timely breast cancer screening at 40 years old by SGM status (**A**) and at 74 years old by SGM status (**B**).

In the model of timely breast cancer screening probability, missing demographic information reduced the analytic sample to 13,891 women. Predicted screening probabilities at age 40 and age 74 were consistently lower for SGM women than for cisgender heterosexual women (38% vs. 47% at age 40 and 69% vs. 77% at age 74; Figure 3A,B). Boxplots also illustrate greater variability in predicted screening probabilities among SGM women, indicating more heterogeneity in screening patterns across both age points.

In adjusted models, several sociodemographic and behavioral factors were associated with screening outcomes; however, these covariates were included as controls and are not the focus of this analysis. Full model estimates are provided in Table 2.

Model-estimated probabilities and absolute risk differences (ARD) for SGM status were calculated. For the past 2-year breast cancer screening, SGM adults had a higher predicted probability of screening than cisgender heterosexual adults (85.0% vs. 83.6%; ARD = 1.4%, 95% CI: 0.2% to 2.6%). For lifetime breast cancer screening, SGM adults had a lower predicted probability compared with cisgender heterosexual adults (88.2% vs. 92.3%; ARD = −4.04%, 95% CI: −5.4% to −2.7%). Although the model-estimated probabilities for mammography were higher than the statewide BRFSS prevalence, this is expected because the regression model was based on a smaller analytic sample restricted to women aged 40–74 with complete covariate data. Individuals who are excluded because of missing data or because they fall outside the screening-eligible age ranges tend to have lower screening rates, which results in higher average predicted probabilities among the remaining analytic sample. In addition, model-estimated probabilities are adjusted for covariates and therefore do not correspond directly to the weighted statewide prevalence. Thus, the differences reflect analytic sample restrictions and model adjustment rather than a discrepancy in the underlying data.

**Figure 4:**
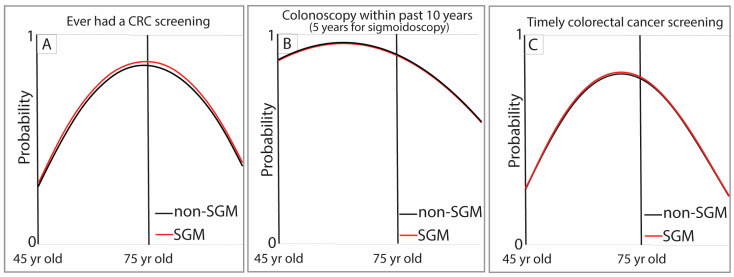
Probability of ever having colorectal cancer (CRC) screening (**A**), in the past ten years for colonoscopy or 5 years for sigmoidoscopy (**B**), and timely CRC screening (**C**) for SGM adults and non-SGM adults.

“Have you ever had a colonoscopy or sigmoidoscopy?” was asked of 35,703 Missourians, aged 45 to 99. After excluding respondents with missing demographic information, the analytic sample included 23,804 adults and 13,205 participants aged 45–75 years who were up to date on CRC screening. The probability of ever having undergone CRC screening increased steadily beginning at age 45, peaked between the mid-60s and early 70s, and declined after age 75 for both SGM adults and cisgender heterosexual adults. Across the age span, SGM adults showed a slightly higher, but similar probability of ever being screened (Figure 4A).

The probability of reporting a colonoscopy in the past 10 years or a sigmoidoscopy in the past five years showed similarly high screening likelihoods for both SGM and cisgender heterosexual adults. Screening probabilities increased through approximately age 60 and declined with advancing age beyond 75 years. The two groups had nearly indistinguishable screening patterns across the guideline-recommended age range (Figure 4B).

Timely colorectal cancer screening followed the same age-related pattern, with increasing probability from age 45, peaking between ages 60 and 70, and tapering off afterward. SGM adults showed slightly higher predicted probabilities of timely CRC screening compared with cisgender heterosexual adults, although the overall pattern remained similar across groups (Figure 4C).

**Figure 5:**
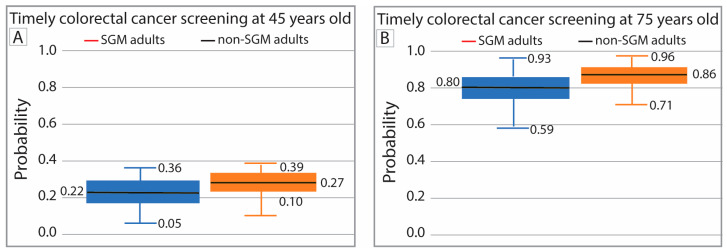
Whisker plot of timely colorectal cancer screening at 45 years old by SGM status (**A**) and at 75 years old by SGM status (**B**).

At age 45, the model estimated that SGM adults had a higher probability of timely CRC screening compared to cisgender heterosexual adults (27% vs. 22%; Figure 5A), although this difference was not statistically significant. Similarly, by age 75, SGM adults were estimated to have a higher probability of timely screening than cisgender heterosexual adults (86% vs. 80%; Figure 5B), but again, this difference did not reach statistical significance.

Adjusted models showed patterns consistent with prior literature, with stronger associations observed for ever having been screened than for up-to-date screening (Table 3). Full covariate estimates are provided for transparency but are not interpreted as independent findings.

For colorectal cancer screening, predicted probabilities were nearly identical for SGM adults and cisgender heterosexual adults for both lifetime screening (67.7% vs. 68.8%; ARD = −1.1%, 95% CI: −3.8% to −1.6%) and recent screening (62.8% vs. 64.6%; ARD = −1.9%, 95% CI: −4.6% to 0.8%), indicating no meaningful differences by SGM status. Although the adjusted regression coefficient for SGM status was positive for ever being screened (Table 3), the model’s predicted probabilities showed only very small differences between groups. This occurs because logistic regression coefficients and predicted probabilities summarize effects in different ways. Small effects on the odds scale often translate into very small or near-zero differences in predicted probabilities. Thus, the results are not contradictory but reflect two valid expressions of the same underlying model.

In all-age models, small group-level differences by SGM status were observed; however, these differences attenuated and were not significant in age-eligible models (primary analyses), indicating that age composition rather than SGM status per se explains the apparent differences (Table 2 and Table 3; Supplementary Tables S1 and S2).

## 4. Discussion

This study finds breast cancer and colorectal cancer screening rates in Missouri to be lower than national averages. Our findings provide evidence of equivalent breast cancer and colorectal cancer screening rates between SGM adults and cisgender heterosexual adults in Missouri when analyses were restricted to screening-eligible ages. Because screening is age-dependent, we emphasized guideline-eligible models. All-age supplemental models are provided for completeness and show patterns consistent with age composition outside the screening window rather than independent disparities (Supplementary Tables S1 and S2). A key strength of this study is the use of a large population-based sample that provides sufficient representation of SGM adults to allow stable, statewide estimates of cancer screening patterns.

Evidence for screening disparity by SGM status is mixed. For breast cancer screening, the Nurses’ Health Study II (NHSII) reported that sexual minority women aged 40–60 years have lower odds of receiving a mammogram in the past two years compared to heterosexual women [40]. In contrast, our findings are consistent with the National Health Interview Study of equivalent rates of mammography between sexual minority and heterosexual women [41]. Similarly, evidence for a disparity in CRC screening by sexual orientation is mixed, depending on the study population and design [42]. Consistent with our results, NHSII found no significant differences in CRC screening rates between sexual minority women and heterosexual women aged 50–60 years [40].

Although CRC screening prevalence increases steadily throughout the recommended ages of 45 to 75 years, it declines among older adults. This pattern suggests that many elderly Missourians have never been screened for CRC. Several factors are likely contributing to this trend. First, CRC screening guidelines were not widely adopted until the mid-1990s, whereas mammography recommendations emerged in the mid-1970s [43,44]. Second, CRC screening is recommended much less frequently, once every ten years for colonoscopy and every five years for sigmoidoscopy, compared to biennial or annual mammography [35,36,37,38]. Finally, CRC screening procedures are often more costly and time-consuming, which may further limit uptake among both SGM older adults and cisgender heterosexuals.

Our age-related patterns in screening probability differed between colorectal and breast cancer and generally mirrored how national guidelines structure screening across the life course. For colorectal cancer, screening probabilities peaked around age 75 and declined sharply thereafter, consistent with guidance recommending routine screening through age 75 and more selective use from ages 76–85 based on life expectancy and prior screening history [45]. In contrast, probabilities for breast cancer screening began to decline earlier, around age 65, but the decline was more gradual. This pattern aligns with more individualized recommendations for mammography in older adults and is supported by major guideline groups, in which decisions after about age 74 depend on comorbidities, functional status, and estimated life expectancy rather than age alone [46,47]. Because breast cancer screening guidelines allow greater flexibility to continue screening in healthy older women than colorectal cancer guidelines, a slower decline in breast cancer screening participation is expected and consistent with observed clinical practice variability.

Within these age-related trends, differences by SGM status were minimal once analyses were confined to guideline-eligible ages. Although all-age predicted probabilities suggested some variation in lifetime screening, these patterns largely reflect age composition and other underlying demographic differences rather than disparities attributable to SGM status itself. In the age-eligible models and in plots comparing probabilities at representative ages, SGM and cisgender heterosexual adults followed remarkably similar trajectories for both breast and colorectal cancer screening. This suggests that, once individuals reach screening-eligible ages and have comparable access to care, SGM adults remain engaged in routine cancer screening at rates that parallel those of cisgender heterosexual adults. This interpretation is further supported by the age-standardized absolute risk differences and the adjusted logistic regression models, both of which demonstrated little to no difference between groups. Although demographic differences existed between groups (Table 1), these population characteristics did not result in meaningful differences in adjusted screening outcomes. In adjusted models, SGM status was not associated with reduced likelihood of up-to-date screening.

These findings underscore an important distinction between patterns observed in age-eligible adults and the dynamics governing initial uptake. Initial uptake of cancer screening (i.e., ever having been screened) is more vulnerable to entrenched access barriers and population-level disparities, reflecting differences accumulated across the life course [48,49]. In contrast, ongoing, up-to-date screening is shaped by additional factors, such as health care continuity, benefit eligibility, particularly as individuals age into Medicare, and longitudinal health beliefs. These influences often differ from the drivers of first-time screening and are further impacted by system-level determinants, including organizational structure and social risks [50,51,52].

These results highlight a need for tailored strategies: efforts to increase first-time screening should focus on overcoming entrenched access barriers, while interventions to support repeat or timely screening must address issues of healthcare continuity, insurance coverage, and culturally competent care. Future research should further disaggregate subgroups and explore intersectional factors, such as race, ethnicity, and rurality, to ensure equitable access and improved outcomes for all Missourians.

Our findings also underscore the importance of distinguishing between apparent disparities observed in unadjusted or all-age samples and those that persist after accounting for guideline-eligible age ranges and sociodemographic differences. Although group-level predicted probabilities suggested modest variation in lifetime breast cancer screening between SGM adults and cisgender heterosexual adults, these differences did not remain once analyses were restricted to screening-eligible ages and adjusted for socioeconomic, geographic, and behavioral factors. This pattern suggests that observed disparities in lifetime screening may reflect differences in underlying population composition, such as age distribution, insurance coverage, or income, rather than inequities attributable to SGM status itself. As such, our age-restricted models indicate that, among adults who have reached the point in the life course when preventive screening is recommended, SGM adults participate in breast and colorectal cancer screening at rates comparable to their cisgender heterosexual counterparts.

However, the similarity in screening rates among age-eligible adults should not obscure the broader structural risks faced by SGM populations. Many SGM adults encounter earlier life course barriers, including stigma, discrimination, fragmented care, and inconsistent insurance coverage, that may delay healthcare engagement or reduce opportunities for preventive services before they reach guideline-eligible ages [18,19,20,24,25,26]. Ensuring equitable cancer prevention, therefore, requires interventions that operate across the continuum of care: strengthening early affirming connections to primary care; expanding insurance and financial protections; supporting culturally competent communication; and improving outreach tailored to SGM communities [20,53,54]. In addition, future work should examine how structural factors intersect with sexual orientation and gender identity, such as racism, rurality, socioeconomic vulnerability, and disability, to influence preventive care access [55,56,57]. Such intersectional approaches will be essential for identifying where disparities emerge and for developing multilevel strategies to promote equitable cancer screening across all populations in Missouri.

One strength of this study is the use of the MO-CLS data to estimate BC and CRC screening prevalence at the county level. Most counties in Missouri have screening rates similar to the statewide average. Notably, the Bootheel region in southeastern Missouri, part of the Mississippi Delta, is recognized for substantial health disparities, including cancer mortality rates that are approximately 16% higher than the national average [58,59]. Of the 22 counties with CRC screening rates below the state average, two are located in the Bootheel region (southeastern corner of Missouri). In contrast, none of the 12 counties with below-average BC screening rates are in the Bootheel. This suggests that programs such as ShowMe Healthy Women, which provides free mammograms to eligible low-income and uninsured women, may be instrumental in reducing geographic disparities in BC screening access [59,60]. In addition, our sensitivity analysis comparing recent and ever screening behavior illustrates the value of examining both lifetime and recent screening outcomes as complementary indicators and provides a nuanced perspective on screening behavior.

This study has several limitations. First, all health and demographic variables were based on self-reports. Self-report is subject to recall and response biases. However, self-report is the most cost-effective and valid means of collecting personal information [61,62]. Further, our study was not an intervention study, so social desirability bias in responses is reduced [63].

Second, our measure of CRC screening was limited by the structure of the MO-CLS, which captured only colonoscopy or sigmoidoscopy utilization. Additional evidence-based CRC screening options, including home-based stool collection (i.e., fecal immunochemical test (FIT) and multi-target stool DNA test (mt-sDNA)), were not captured by the MO-CLS. This limits comparability with national surveillance systems that include these CRC screening modalities.

Third, breast cancer screening guidelines vary across professional organizations. The American Cancer Society, American College of Radiology, and National Comprehensive Cancer Network recommend annual BC screenings for women at average risk starting at 40 years of age, whereas the US Preventive Services Task Force recommends biennial mammography screening at 40 years of age [35,36,37,38]. In this regard, BC screening frequency for those aged 40–49 years is dependent on which agency’s recommendation the clinician–patient dyad follows.

Fourth, the two-stage modeling strategy used to estimate timely screening required restricting the second-stage model to individuals who had ever been screened. This may introduce selection bias if individuals who initiate screening differ systematically from those who have never been screened in ways not fully captured in the data. Estimates of timely screening should therefore be interpreted with this limitation in mind.

Finally, generational differences in willingness to publicly identify as an SGM individual, especially within the context of a telephone survey, may have led to the age distribution observed among SGM participants [64]. This, in turn, could influence estimates of screening patterns across the life course.

## 5. Conclusions

In summary, our findings suggest that SGM status is not a major determinant of guideline-eligible breast cancer or colorectal cancer screening in Missouri; instead, long-standing socioeconomic and geographic disparities continue to shape who is screened. Strengthening community-based outreach for underserved groups, reducing cost and access barriers, continuing investment in evidence-based screening programs, and ensuring culturally competent care remain essential steps toward improving screening for all populations statewide.

## Figures and Tables

**Table 1:** Comparison of characteristics of county-level study participants by sexual and gender minority (SGM) status.

	SGM n (%) (n = 2801)	Cisgender Heterosexual n (%) (n = 48,257)	*p*-Value	Cramér’s V
Age groups				
18–39 years	1426 (51.5)	9684 (20.4)	<0.0001	0.173
40–64 years	767 (27.7)	18,294 (38.6)		
65 and older	575 (20.8)	19,460 (41.0)		
Total	2768	47,438		
				
Race/Ethnicity				
White non-Hispanic	2164 (78.7)	40,814 (86.5)	0.0011	0.08
Black non-Hispanic	203 (7.4)	3345 (7.1)		
Asian	47 (1.7)	482 (1.0)		
American Indian/Alaskan Native	36 (1.3)	428 (0.9)		
* Another race	18 (0.9)	52 (0.1)		
Multi-race	90 (3.3)	710 (1.5)		
Hispanic	183 (6.7)	1372 (2.9)		
Total	2749	47,203		
				
Rural–urban commuting area (RUCA)				
Isolated	360 (13.1)	9729 (20.8)	<0.0001	0.056
Small rural	384 (14.0)	6621 (14.1)		
Large rural	410 (14.9)	8470 (18.1)		
Urban	1593 (58.0)	22,054 (47.0)		
Total	2747	46,874		
				
Educational attainment				
Less than high school	224 (8.0)	3048 (6.3)	0.8571	0.019
High school degree or GED †	871 (31.2)	16,167 (33.7)		
Some college	890 (31.9)	14,763 (30.7)		
4-year college degree	499 (17.9)	8476 (17.6)		
Postgraduate	310 (11.1)	5589 (11.6)		
Total	2794	48,043		
				
Income				
<$25,000	623 (28.9)	7350 (20.7)	<0.0001	0.057
$25,000–$49,999	578 (26.8)	8889 (25.0)		
$50,000–$74,999	370 (17.2)	6139 (17.3)		
$75,000–$99,999	250 (11.6)	5394 (15.2)		
$100,000 or more	335 (15.5)	7756 (21.8)		
Total	2156	47,768		
				
Employment				
Employed	1634 (53.2)	23,360 (48.9)	<0.0001	0.105
Unemployed, Student, or Homemaker	402 (14.5)	3198 (6.7)		
Retired	502 (18.1)	17,883 (37.4)		
Unable to work	237 (8.5)	3327 (7.0)		
Total	2775	47,768		
				
Insurance				
Employer (private)	1489 (53.2)	23,825 (49.4)	<0.0001	0.084
Medicare/Medigap	609 (21.7)	17,334 (35.9)		
Medicaid or other assistance	307 (11.0)	2722 (5.6)		
Unknown	396 (14.1)	4376 (9.1)		
Total	2801	48,257		
				
Marital Status				
Single/Never Married	1274 (45.8)	7599 (15.9)	<0.0001	0.181
Married	847 (30.5)	26,199 (54.7)		
Divorced, Separated, or Widowed	660 (23.7)	14,091 (29.4)		
Total	2781	47,889		
				
Home Ownership				
Yes	1489 (53.5)	37,311 (77.9)	<0.0001	0.131
Total	2784	47,897		
				
† MET Physical Activity guidelines				
Yes	2006 (71.8)	34,009 (70.6)	0.1847	0.006
Total	2793	48,152		
				
Smoking Status				
Never	1553 (55.7)	26,026 (55.6)	0.0083	0.03
Former	670 (24.0)	13,235 (28.3)		
Current	563 (20.2)	7516 (16.1)		
Total	2786	46,777		

* Another race includes Pacific Islander, Native Hawaiian, Guamanian or Chamorro, Samoan, Other Pacific Islander, and the Other category, as written on the survey. † GED: General Equivalency Diploma; MET: metabolic equivalent of task. The denominator excludes respondents with refused/missing responses.

**Table 2:** Quadratic regression models for ever having had a mammogram and having had a mammogram within the past two years, 2022 County-Level Study, Missouri (women 40–74 years of age only).

	Ever Had a Breast Cancer Screening (n = 11,092)			Within Past Two Years (n = 10,062)		
	Estimate	95% CI	*p*-Value	Estimate	95% CI	*p*-Value
**Intercept**	**(1.24)**	**(1.42)–(1.06)**	**<0.0001**	**1.29**	**1.001–1.578**	**<0.0001**
**Age**	**0.073**	**0.066–0.079**	**<0.0001**	**(0.015)**	**(0.026)–(0.005)**	**0.003**
**Age squared**	**(0.0006)**	**(0.0006)–(0.0005)**	**<0.0001**	**(0.0001)**	**0.0000–0.0002**	**0.006**
Sexual and gender minority	(0.001)	(0.022)–(0.019)	0.9066	0.014	(0.017)–0.045	0.383
Rural–urban commuting area (RUCA)						
Urban	reference					
Large rural	(0.003)	(0.020)–0.013	0.709	0.009	(0.015)–0.034	0.457
Small rural	(0.022)	(0.039)–(0.004)	0.0157	(0.019)	(0.045)–0.008	0.167
*** Isolated***	(0.019)	(0.039)–0.001	0.0602	**(0.040)**	**(0.070)–(0.010)**	**0.009**
Race-ethnicity						
White/Non-Hispanic	reference					
** Black/Non-Hispanic**	**0.0345**	**0.018–0.051**	**<0.0001**	**0.093**	**0.069–0.117**	**<0.0001**
Another race/Non-Hispanic	(0.022)	(0.053)–0.008	0.153	(0.015)	(0.062)–0.031	0.510
*** Multi-race/Non-Hispanic***	**(0.097)**	**(0.128)–(0.065)**	**<0.0001**	0.038	(0.012)–0.087	0.135
Hispanic	(0.005)	(0.038)–0.027	0.757	0.030	(0.019)–0.079	0.228
Education attainment						
Less than a 4-year degree	reference					
** Bachelor’s or higher**	**0.020**	**0.008–0.031**	**0.007**	**(0.027)**	**(0.044)–(0.010)**	**0.002**
Income						
** Less than $25,000**	**(0.075)**	**(0.098)–(0.052)**	**<0.0001**	**(0.144)**	**(0.178)–(0.110)**	**<0.0001**
** $25,000–49,999**	**(0.077)**	**(0.094)–(0.060)**	**<0.0001**	**(0.056)**	**(0.081)–(0.031)**	**<0.0001**
** $50,000–74,999**	**(0.049)**	**(0.064)–(0.033)**	**<0.0001**	**(0.042)**	**(0.065)–(0.020)**	**0.0003**
*** $75,000–99,999***	**(0.022)**	**(0.036)–(0.007)**	**0.004**	(0.015)	(0.036)–0.007	0.1785
$100,000 or higher	Reference					
Employment status						
Employed full- or part-time	reference					
*** Unemployed, Homemaker, Student***	(0.002)	(0.025)–0.020	0.832	**(0.094)**	**(0.128)–(0.059)**	**<0.0001**
** Retired**	**0.039**	**0.023–0.056**	**<0.0001**	0.024	(0.000)–0.048	0.052
** Unable to Work**	**0.047**	**0.025–0.069**	**<0.0001**	0.010	(0.024)–0.043	0.576
Insurance						
Employer (private)	reference					
Medicare or Medigap	0.019	0.002–0.037	0.031	0.020	(0.006)–0.045	0.134
Medicaid or other assistance	(0.033)	(0.059)–(0.007)	0.012	0.018	(0.022)–0.058	0.390
Marital status						
Single/never married	Reference					
Married	0.013	(0.004)–0.030	0.149	(0.027)	(0.053)–(0.001)	0.040
**Separated, Widowed, Divorced**	**0.034**	**0.017–0.051**	**0.0001**	**(0.055)**	**(0.081)–(0.029)**	**<0.0001**
Home ownership						
***Own home***	0.018	0.003–0.032	0.015	**0.033**	**0.011–0.055**	**0.003**
***Exercise within the past 30 days***	(0.006)	(0.018)–0.005	0.272	**0.063**	**0.046–0.080**	**<0.0001**
Smoking status						
Never	reference					
*** Former***	**(0.018)**	**(0.030)–(0.006)**	**0.002**	0.007	(0.010)–0.024	0.445
** Current**	**(0.034)**	**(0.049)–(0.020)**	**<0.0001**	**(0.075)**	**(0.097)–(0.053)**	**<0.0001**

Denominator excludes respondents with refused/missing responses; percentages are weighted to population characteristics. Another race includes Asian Non-Hispanic, American Indian or Alaskan Native Non-Hispanic, Pacific Islander, Native Hawaiian, Guamanian or Chamorro, Samoan, Other Pacific Islander, and the Other category, as written on the survey. Negative numbers for estimates and confidence intervals are indicated as ( ); bold black text if significant at both time periods and bold italic text if significant at one time period; *p* < 0.01 or less. See Supplementary Table S1 for all-age models. Differences reflect broader sample heterogeneity beyond guideline-eligible age groups.

**Table 3:** Quadratic regression model of predictors of ever having had colorectal cancer screening or colonoscopy within the past 10 years or sigmoidoscopy within the past 5 years, 2022 County-Level Study, Missouri, 45–75 years of age.

	Ever Had Colorectal Cancer Screening (n = 19,046)			Colonoscopy Within Past 10 Years or Sigmoidoscopy Within Past 5 Years (n = 13,205)		
	Estimate	95% CI	*p*-Value	Estimate	95% CI	*p*-Value
***Intercept***	**(5.019)**	**(5.34)–(4.70)**	**<0.0001**	(0.003)	(0.246)–0.240	0.983
**Age**	**0.173**	**0.161–0.183**	**<0.0001**	**0.030**	**0.022–0.038**	**<0.0001**
**Age squared**	**(0.0013)**	**(0.0014)–(0.0011)**	**<0.0001**	**(0.0002)**	**(0.0003)–(0.0002)**	**<0.0001**
Sex						
Male			reference			
Female	0.012	(0.001)–0.024	0.0728	0.002	(0.007)–0.010	0.6945
***Sexual and gender minority***	**0.097**	**0.069–0.125**	**<0.0001**	0.013	(0.006)–0.032	0.1817
Rural–urban commuting area (RUCA)						
Urban	reference					
*** Large rural***	**(0.027)**	**(0.047)–(0.007)**	**0.0084**	0.018	0.004–0.032	0.0111
*** Small rural***	**(0.033)**	**(0.055)–(0.012)**	**0.0028**	(0.008)	(0.024)–0.007	0.2850
*** Isolated***	**(0.050)**	**(0.074)–(0.027)**	**<0.0001**	(0.005)	(0.011)–0.021	0.5461
Race-ethnicity						
White/Non-Hispanic	Reference					
** Black/Non-Hispanic**	**0.0391**	**0.018–0.060**	**0.0003**	**0.036**	**0.021–0.150**	**<0.0001**
*** Another race/Non-Hispanic***	(0.046)	(0.090)–(0.002)	0.0396	**0.056**	**0.022–0.089**	**0.0012**
Multi-race Non-Hispanic	(0.041)	(0.081)–(0.002)	0.0398	(0.002)	(0.030)–0.025	0.8621
Hispanic	(0.021)	(0.062)–0.020	0.3079	0.021	(0.009)–0.051	0.1630
Education attainment						
Less than a 4-year degree	reference					
*** Bachelor’s or higher***	**0.056**	**0.042–0.070**	**<0.0001**	0.010	0.001–0.019	0.0386
Income						
*** Less than $25,000***	**(0.149)**	**(0.175)–(0.123)**	**<0.0001**	(0.005)	(0.024)–0.013	0.5501
** $25,000–49,999**	**(0.070)**	**(0.090)–(0.050)**	**<0.0001**	**(0.020)**	**(0.033)–(0.006)**	**0.0047**
$50,000–74,999	(0.023)	(0.042)–(0.004)	0.0181	0.011	(0.002)–0.024	0.1103
*** $75,000–99,999***	**(0.040)**	**(0.058)–(0.022)**	**<0.0001**	0.010	(0.003)–0.022	0.1299
$100,000 or higher	Reference					
Employment status						
Employed full- or part-time	reference					
*** Unemployed, Student, or Homemaker***	**0.064**	**0.030–0.020**	**0.0002**	(0.011)	(0.035)–0.013	0.3553
*** Retired***	**0.093**	**0.074–0.111**	**<0.0001**	0.004	(0.008)–0.016	0.5099
*** Unable to Work***	**0.152**	**0.125–0.179**	**<0.0001**	(0.017)	(0.035)–0.001	0.0673
Insurance						
Employer (private)	reference					
Medicare or Medigap	(0.011)	(0.030)–0.009	0.2747	0.016	0.004–0.028	0.0118
*** Medicaid or other assistance***	**(0.054)**	**(0.086)–(0.023)**	**0.0006**	0.027	0.004–0.049	0.0177
Marital status						
Single/never married	Reference					
** Married**	**0.093**	**0.072–0.115**	**<0.0001**	**0.024**	**0.008–0.040**	**0.0029**
*** Separated, Widowed, Divorced***	**0.096**	**0.074–0.119**	**<0.0001**	0.008	(0.008)–0.024	0.3153
Home ownership						
Own home	(0.002)	(0.020)–0.016	0.7958	(0.012)	(0.025)–0.001	0.0611
Exercise within the past 30 days	(0.010)	(0.024)–0.004	0.1515	(0.001)	(0.010)–0.009	0.8791
Smoking status						
Never	reference					
Former	0.018	0.030–0.006	0.0143	0.006	(0.003)–0.015	0.2098
*** Current***	**(0.038)**	**(0.055)–(0.020)**	**<0.0001**	(0.007)	(0.019)–0.005	0.2652

Denominator excludes respondents with refused/missing responses. Percentages are weighted to population characteristics. Another race includes Asian Non-Hispanic, American Indian or Alaskan Native Non-Hispanic, Pacific Islander, Native Hawaiian, Guamanian or Chamorro, Samoan, Other Pacific Islander, and the Other category, as written on the survey. Negative numbers for estimates and confidence intervals are indicated as ( ); bold black text if significant at both time periods and bold italics text if significant at one time period; *p* < 0.01 or less. See Supplementary Table S2 for all-age models. Differences reflect broader sample heterogeneity beyond guideline-eligible age groups.

## Data Availability

Aggregate MO-CLS data is available upon request by contacting anthony.belenchia@health.mo.gov.

## Supplementary Materials

The following supporting information can be downloaded at: https://www.mdpi.com/article/10.3390/cancers18050729/s1, Table S1: Log-normal regression model and quadratic regression model for ever had mammogram or mammogram within past two years, 2022 County Level Study, Missouri all ages (18–99 years); Table S2: Quadratic regression model of predictors of ever having had colorectal cancer screening or colonoscopy within past 10 years or sigmoidoscopy within past 5 years, 2022 County Level Study, Missouri, 45–99 years of age.
