# Supplementary material for: Comparing Sexual and Gender Minority and Cisgender Heterosexual Missourians’ Breast and Colorectal Cancer Screening Prevalence: The 2022 Missouri County-Level Study
Source: Cancers (Basel). 2026 Feb 24;18(5):729. doi: 10.3390/cancers18050729 (PMC12984082; doi:10.3390/cancers18050729)
Supplement: Supplementary file 1 [file cancers-18-00729-s001.zip › cancers-4131077-supplementary.pdf]

Supplement Table S1. Log-normal regression model and quadratic regression model for ever had mammogram or mammogram within past two years, 2022 County Level Study, Missouri all ages (18-99 years)

	Ever had a breast cancer screening			Within past two years		
	Estimate	95% CI	p-value	Estimate	95% CI	p-value
**Intercept**	**(11.36)**	**(11.86)-(10.86)**	**<0.0001**	**0.237**	**0.156-0.318**	**<0.0001**
**Age**				**0.024**	**0.021-0.026**	**<0.0001**
**Age squared**				**(0.0002)**	**(0.0002)-(0.0002)**	**<0.0001**
**Log10(Age)**	**7.50**	**7.18-7.82**	**<0.0001**			
**Sexual and gender minority**	**(0.138)**	**(0.243)-(0.033)**	**0.0101**	**(0.040)**	**(0.068)-(0.01.)**	**0.004**
Rural-urban commuting area (RUCA)						
Urban			reference			
Large rural	0.024	(0.088)-0.136	0.673	(0.128)	(0.035)-0.009	0.257
Small rural	(0.092)	(0.221)-0.037	0.164	(0.025)	(0.050)-(0.001)	0.043
Isolated	(0.107)	(0.253)-0.039	0.152	**(0.045)**	**(0.072)-(0.017)**	**0.001**
Race-ethnicity						
White/Non-Hispanic			reference			
**Black/Non-Hispanic**	**0.222**	**0.112-0.332**	**<.0001**	**0.075**	**0.053-0.096**	**<.0001**
Another races/Non-Hispanic	(0.128)	(0.308)-0.052	0.165	(0.04)	(0.084)-0.003	0.068
Multi-race/Non-Hispanic	(0.778)	(0.377)-0.022	0.082	(0.006)	(0.049)-0.036	0.769
Hispanic	0.105	(0.074)-0.283	0.250	(0.027)	(0.070)-0.017	0.233
Education attainment						
Less than 4 year degree			reference			
Bachelor's or higher	(0.064)	(0.144)-0.016	0.117	**(0.023)**	**(0.039)-(0.007)**	**0.004**
Income						
**Less than $25,000**	**(0.334)**	**(0.491)-(0.171)**	**<0.0001**	**(0.124)**	**(0.153)-(0.095)**	**<0.0001**
**$25,000- 49,999**	**(0.287)**	**(0.402)-(0.171)**	**<0.0001**	**(0.044)**	**(0.066)-(0.021)**	**0.000**
**$50,000- 74,999**	**(0.337)**	**(0.441)-(0.234)**	**<0.0001**	**(0.036)**	**(0.058)-(0.015)**	**0.001**
$75,000- 99,999	**(0.208)**	**(0.310)-(0.106)**	**<0.0001**	(0.001)	(0.022)-0.019	0.886
$100,000 or higher			reference			
Employment status						
Employed full- or part-time			reference			
Unemployed, Homemaker, Student	0.030	(0.087)-0.147	0.618	**(0.053)**	**(0.083)-(0.023)**	**0.001**
Retired	0.031	(0.134)-0.197	0.712	**0.035**	**0.012-0.058**	**0.002**
Unable to Work	0.181	0.000-0.361	0.050	(0.008)	(0.040)-0.023	0.608
Insurance						
Employer (private)			reference			
Medicare or Medigap	(0.049)	(0.200)-0103	0.527	**0.046**	**0.024-0.067**	**<0.0001**
Medicaid or other assistance	0.029	(0.120)-0.178	0.702	0.017	(0.011)-0.056	0.194
Marital status						
Single/never married			reference			

**Married**	**(0.216)**	**(0.313)-(0.119)**	**<0.0001**	**(0.051)**	**(0.074)-(0.028)**	**<0.0001**
Separated, Widowed, Divorced	(0.060)	(0.170)-0.050	0.285	**(0.084)**	**(0.107)-(0.061)**	**<0.0001**
Home ownership						
Own home	0.045	(0.042)-0.133	0.309	**0.032**	**0.013-0.051**	**0.001**
Exercise within past 30 days	0.028	(0.057)-0.113	0.517	**0.056**	**0.040-0.071**	**<0.0001**
Smoking status						
Never			reference			
Former	(0.067)	(0.154)-0.020	0.130	0.008	(0.008)-0.023	0.346
Current	(0.076)	(0.179)-0.027	0.150	**(0.072)**	**(0.092)-(0.051)**	**<0.0001**

Denominator excludes respondents with refused/missing responses
Percentages are weighted to population characteristics
Another race includes Asian Non-Hispanic, American Indian or Alaskan Native Non-Hispanic, Pacific Islander, Native Hawaiian, Guamanian or Chamorro, Samoan, Other Pacific Islander and Other category as written on the survey
Negative numbers for estimates and confidence intervals are indicated as ( )
Bold black text if significant at both time periods and bold italics text if significant at one time period; p < 0.01 or less

Supplement Table S2. Quadratic regression model of predictors of ever having had colorectal cancer screening or colonoscopy within past 10 years or sigmoidoscopy within past 5 years, 2022 County Level Study, Missouri, 45-99 years of age

	Ever had colorectal cancer screening			Colonoscopy within past 10 years or sigmoidoscopy within past 5 years		
	Estimate	95% CI	p-value	Estimate	95% CI	p-value
Intercept	**(3.369)**	**(3.527)-(3.210)**	**<0.0001**	(0.047)	(0.184)-0.090	0.499
**Age**	**0.115**	**0.110-0.119**	**<0.0001**	**0.032**	**0.028-0.036**	**<0.0001**
**Age squared**	**(0.001)**	**(0.001)-(0.001)**	**<0.0001**	**(0.0003)**	**(0.0003)-(0.0002)**	**<0.0001**
Sex						
Male			reference			
Female	**0.018**	**0.007-0.029**	**0.002**	(0.007)	(0.016)-0.001	0.077
Sexual and gender minority	**0.081**	**0.056-0.106**	**<0.0001**	(0.003)	(0.022)-0.015	0.736
Rural-urban commuting area (RUCA)						
Urban			reference			
Large rural	**(0.031)**	**(0.048)-(0.013)**	**0.001**	0.005	(0.008)-0.019	0.443
Small rural	**(0.041)**	**(0.060)-(0.022)**	**<0.0001**	(0.008)	(0.023)-0.006	0.265
Isolated	**(0.062)**	**(0.083)-(0.042)**	**<0.0001**	0.001	(0.015)-0.016	0.925
Race-ethnicity						
White/Non-Hispanic			reference			
**Black/Non-Hispanic**	**0.029**	**0.010-0.048**	**0.0002**	**0.044**	**0.030-0.058**	**<0.0001**
**Another race/Non-Hispanic**	**(0.080)**	**(0.189)-(0.040)**	**<0.0001**	**0.063**	**0.029-0.096**	**0.0002**
Multi-race/Non-Hispanic	(0.029)	(0.065)-0.006	0.102	**(0.049)**	**(0.075)-(0.022)**	**0.0004**
Hispanic	(0.024)	(0.062)-0.014	0.222	0.013	(0.017)-0.043	0.407

Education attainment						
Less than 4 year degree			reference			
**Bachelor's or higher**	**(0.046)**	**0.033-0.058**	**<0.0001**	**0.014**	**0.004-0.023**	**0.004**
Income						
Less than $25,000	**(0.151)**	**(0.173)-(0.128)**	**<0.0001**	0.010	(0.007)-0.027	0.246
$25,000- 49,999	**(0.078)**	**(0.096)-(0.060)**	**<0.0001**	(0.005)	(0.018)-0.008	0.427
$50,000- 74,999	**(0.032)**	**(0.049-(0.014)**	**0.0004**	0.013	0.000-0.026	0.044
$75,000- 99,999	**(0.040)**	**(0.056)-(0.023)**	**<0.0001**	0.006	(0.006)-0.018	0.333
$100,000 or higher			reference			
Employment status						
Employed full- or part-time			reference			
Unemployed, Homemaker, Student	**0.070**	**0.039-0.101**	**<0.0001**	(0.006)	(0.029)-0.018	0.646
Retired	**0.072**	**0.056-0.088**	**<0.0001**	0.009	(0.003)-0.020	0.134
**Unable to Work**	**0.165**	**0.141-0.190**	**<0.0001**	**(0.027)**	**(0.044)-(0.009)**	**0.004**
Insurance						
Employer (private)			reference			
**Medicare or Medigap**	**(0.040)**	**(0.056)-(0.025)**	**<0.0001**	**0.015**	**0.004-0.026**	**0.006**
**Medicaid or other assistance**	**(0.062)**	**(0.090)-(0.033)**	**<0.0001**	**0.029**	**0.007-0.051**	**0.010**
Marital status						
Single/never married			reference			
**Married**	**0.079**	**0.059-0.099**	**<0.0001**	**0.025**	**0.009-0.041**	**0.002**
Separated, Widowed, Divorced	**0.095**	**0.074-0.115**	**<0.0001**	0.011	(0.005)-0.027	0.171
Home ownership						
Own home	(0.002)	(0.018)-0.014	0.801	(0.012)	(0.024)-0.000	0.050
Exercise within past 30 days	(0.008)	(0.020)-0.005	0.216	(0.003)	(0.012)-0.006	0.514
Smoking status						
Never			reference			
Former	0.008	(0.004)-0.020	0.207	**0.015**	**0.006-0.023**	**0.001**
Current	**(0.038)**	**(0.054)-(0.022)**	**<0.0001**	(0.002)	(0.015)-0.010	0.713

Denominator excludes respondents with refused/missing responses
Percentages are weighted to population characteristics
Another race includes Asian Non-Hispanic, American Indian or Alaskan Native Non-Hispanic, Pacific Islander, Native Hawaiian, Guamanian or Chamorro, Samoan, Other Pacific Islander and Other category as written on the survey
Negative numbers for estimates and confidence intervals are indicated as ( )
Bold black text if significant at both time periods and bold italics text if significant at one time period; $p < 0.01$ or less.
